# Effect of Maternal Lipopolysaccharide Administration on the Development of Dopaminergic Receptors and Transporter in the Rat Offspring

**DOI:** 10.1371/journal.pone.0054439

**Published:** 2013-01-17

**Authors:** Moogeh Baharnoori, Sanjeev K. Bhardwaj, Lalit K. Srivastava

**Affiliations:** Departments of Psychiatry and Neurology and Neurosurgery, McGill University, and Douglas Mental Health University Institute, Montreal, Quebec, Canada; University of Minnesota, United States of America

## Abstract

Epidemiological evidence supports that maternal infection during gestation are notable risk factors for developmental mental illnesses including schizophrenia and autism. In prenatal lipopolysaccharide (LPS) model of immune activation in rats, the offspring exhibit significant impairments in behaviors mediated by central dopamine (DA) system. This study aimed to examine the temporal and regional pattern of postnatal DA development in the male offspring of pregnant Sprague-Dawley rats administered with 100 µg/kg LPS or saline at gestational days 15/16. Using ligand autoradiography, D1 and D2 dopamine receptors (D1R, D2R) and dopamine transporter (DAT) binding levels were measured in the prefrontal cortex (PFC) and sub cortical regions (dorsal striatum and nucleus accumbens core and shell) at pre pubertal (P35) and post pubertal ages (P60). We found a significant decrease in D2R ligand [^3^H] YM-90151-2 binding in the medial PFC (mPFC) in prenatal LPS-treated animals at P35 and P60 compared to respective saline groups. The decrease in D2R levels was not observed in the striatum or accumbens of maternal LPS-treated animals. No significant changes were observed in [^3^H] SCH23390 binding to D1R. However, the level of [^125^I] RTI-121 binding to DAT was selectively reduced in the nucleus accumbens core and shell at P35 in the prenatal LPS group. Immunohistochemical analysis showed that number of D2R immunopositive cells in infralimbic/prelimbic (IL/PL) part of mPFC was significantly reduced in the LPS group at P60. Prenatal LPS treatment did not significantly affect either the total number of mature neurons or parvalbumin (PV)-immunopositive interneurons in this region. However the number of PV and D2R co-labeled neurons was significantly reduced in the IL/PL subregion of PFC of LPS treated animals. Our data suggests D2R deficit in the PFC and PV interneurons may be relevant to understanding mechanisms of cortical dysfunctions described in prenatal infection animal models as well as schizophrenia.

## Introduction

Etiology of complex mental disorders of aberrant neurodevelopment such as schizophrenia and autism remains poorly understood. Familial aggregation and high levels of heritability of these disorders suggests important contribution of genetic factors 1,2. At the same time epidemiological studies provide substantial evidence that pre and/or perinatal environmental risk factors, possibly in conjunction with genetic vulnerability, contribute significantly in triggering the developmental cascade of these disorders [3], [4]. Among environmental risk factors, several studies confirm that maternal exposure to viral and bacterial pathogens and maternal immune activation are significantly associated with increased incidence of schizophrenia and autism [5]–[8]. Animal studies using models of immune activation mimicking viral and bacterial infections provide evidence of a causal link as the offspring of immune-challenged pregnant rodents show behavioral, cognitive and cellular abnormalities reminiscent of those reported in these disorders [9]–[11]. These studies commonly employ systemic maternal administration of either bacterial endotoxin lipopolysaccaride (LPS) or polyinosinic:polycytidylic acid (poly I:C) to mimic bacterial and viral immune responses respectively. While there are some differences in the behavioral and neural outcome in the offspring following maternal LPS and poly (I:C) administrations (in keeping with differences in their mechanisms of action) and gestational timing of exposure [12], [13], it is often observed that maternal immune activations elicit neurodevelopmental abnormalities, alterations in markers of GABA, glutamate and central dopamine (DA) system in the offspring [14]–[17].

Many of the behavioral abnormalities reported in rodent models of prenatal infection appear to have a close relationship with imbalances in mesolimbic and mesocortical DA pathways. For example, prenatal administration of LPS in rats causes significant deficits in prepulse inhibition of acoustic startle and increases in amphetamine induced locomotion in the adult offspring [12], [18]–[23]. At the cellular level, maternal immune activation by poly (I:C) or LPS leads to increased number of fetal mesencephalic DA neurons as well as postnatal increase in the expression tyrosine hydroxylase in the midbrain and striatum [18], [20], [21]. Reports also show alterations in DA receptors in cortical and subcortical brain regions following prenatal administration of polyI:C [21], [24].

The synaptic actions of DA are mediated by two classes of DA receptors, the mainly post-synaptic D1R (comprising D1and D5Rs) and pre and post-synaptic D2R (comprising D2, D3 and D4Rs) [25]. D1 and D2 receptors are broadly distributed in the brain with most prominent expression in the striatum and mesolimbic DA regions such as nucleus accumbens (Nacc). D1 and D2 receptors are also expressed in deep layers of the prefrontal cortex (PFC), on both principal pyramidal neurons as well as classes of interneurons [26], [27]. The principal mechanism for limiting DA synaptic action involves reuptake of released DA into presynaptic terminals by the DA transporter (DAT) expressed on DA terminals [28]. DA system is considered to be a key player in neurodevelopmental psychiatric illnesses such as schizophrenia [29], [30] as well as autism [31], [32]. It is widely believed that positive symptoms of schizophrenia may result from an excess of DAergic neurotransmission in the striatal/mesolimbic regions while DA deficits in the PFC may contribute to cognitive impairments.

In the present study, using rats that were exposed to LPS during mid-gestation, we investigated the expression of D1 and D2 receptors and DAT in the PFC, dorsal striatum and Nacc at pre and postpubertal ages [postnatal days (P) 35 and 60] by ligand autoradiography. D2R binding was found to be reduced in the PFC, and we confirmed it using D2R immunohistochemistry. Stereological counting of total neuronal population and parvalbumin (PV) containing interneurons in the PFC did not reveal any differences between maternal LPS and saline-treated rats. However, the proportion of PV-immunopositive neurons co-expressing D2R was significantly reduced in the LPS group. Given the emerging idea of the role of cortical interneurons in neuropsychiatric disorders such as schizophrenia [33], [34], we believe our data may help better understand the mechanisms of cortical dysfunctions following maternal infection in animals as well as in humans.

## Materials and Methods

### Ethics Statement

All procedures were performed in accordance with the guidelines established by the Canadian Council on Animal Care, and were approved by the McGill University Animal Care Committee.

### Animals

Timed Pregnant Sprague-Dawley rats (Charles River Laboratory, Quebec) were shipped to our animal facility at gestational day (GD) 9 and were individually housed in a temperature and humidity-controlled room on a 12 h light/dark cycle with *ad libitum* access to food and water. The animals were injected intraperitoneally (ip) with 100 μg/kg of LPS (from *E. coli* serotype 0111:B4, L-2630, Sigma, Canada) or saline once daily, at GD 15 and 16 (n = 5–6 per group). On the day of birth, the pups were cross-fostered with surrogate dams in mixed litters (litter size = 8–9). Only male pups were retained for the study.

### Quantitative Autoradiography

Rats were killed by rapid decapitation at PD35 (*n* = 6–7) or at PD60 (*n* = 5–6), brains were rapidly removed, frozen in a 2 methyl-isobutane cooled on dry ice (−40°C) and then stored at −80°C. Frozen rat brains were sectioned at 20 µm thickness in the coronal plane using a cryostat (Microm, HM500 M). Sections (3sections/slide) were mounted on the microslides (Snow-coat extra, Surgipath) and stored at −80°C until the day of the experiment. Brain sections at the level of the prefrontal cortex (plate 7–10) and dorsal striatum and Nacc (plate 14–18) [35] were selected for ligand autoradiography experiments. The autoradiography procedures were essentially as described by us previously [36], [37].

### D1-receptor autoradiography

The sections were first pre incubated for 10 min in the assay buffer containing 50 mM Tris-HCI, 154 mM NaCl, 1 mM EDTA, and 0.1% bovine serum albumin (pH:7.4) at room temperature. Sections were then incubated in the same buffer containing 2 nM [^3^H] SCH23390 (Perkin Elmer) for 90 minutes at room temperature. Non specific binding was determined on adjacent sections collected on fresh slides by adding 1 µM (+) butaclamol (Sigma-Aldrich) and 30 nM ketanserin (Sigma-Aldrich) (to mask binding to 5-HT2 sites). The incubation was terminated by dipping the slides in ice-cold buffer followed by 2 times wash (10 minutes each) in the same buffer. Slides were dried at room temperature and exposed to 3[H]-sensitive Hyperfilm (Kodak) along with microscale-calibrated tritium standards for 4 weeks for limbic region and 6 weeks for prefrontal cortex.

### D2-receptor autoradiography

The sections were pre incubated in 50 mM Tris-HCI (pH: 7.4) containing 120 mM NaCl, 1 mM EDTA, 5 mM KCl, 1.5 mM CaCl_2_ and 2 mM MgCl_2_ for 10 min. These sections were then incubated in the same buffer containing 1 nM [^3^H] YM-90151-2 (Perkin Elmer) with or without 1 µM (+) butaclamol in order to asses total and non specific bindings respectively. 50 nM 8-OH-DPAT (Sigma-Aldrich) was added to mask the possible binding to 5HT-1A sites.

### Dopamine transporter (DAT) autoradiography

Sections were first pre incubated in 50 mM Tris-HCI containing 100 mM NaCl and 2 mM KCl (pH:7.4) for 10 min. Then incubation was done in the same buffer containing 50 pM [^125^I] RTI-121 (Perkin Elmer), 0.025% bovine serum albumin and 1 µM fluoxetine HCl (Sigma-Aldrich). Non-specific binding was determined using 100 µM nomifensine (Sigma-Aldrich). The sections were opposed to BioMax MR film for 5 days alongside microscale-calibrated iodinated standards.

### Analysis

The films were analyzed using a computerized image-analysis system (MCID-4, Imaging Research, St. Catherines, Ontario, Canada). The optical density of the areas of interest was converted to fmol mg^−1^ protein by comparison with optical densities of known standards from Amersham International. Specific binding was obtained by subtracting non-specific binding from total receptor binding. Binding data in the PFC and striatum/Nacc regions were analyzed separately as the sections were exposed with separate set of films for different lengths of time. Nacc analysis included both core and shell sub-territories. PFC analysis included sub- regions within the mPFC (i.e., infralimbic/prelimbic (IL/PL), cingulate cortex (CG1) as well as secondary motor cortex (M2). Data were analyzed statistically by the two-way analysis of variance (ANOVA) followed by *post-hoc* Bonferroni test, P<0.05 considered significant.

### Immunohistochemistry

LPS or saline treated offspring (P35 or P60, *n* = 5–6, 1-2pups/dam) were anesthetized with Ketamine-Xylazine mixture (100 mg/kg Ketamine, 0.8 mg/kg xylazine) and transcardially perfused with 4% paraformaldehyde. The fixed brains were sectioned at 40 µm on a coronal plane using a vibratome (VT1000S, Leica). The free floating sections were stored in antifreeze solution at −20°C till further processing. For immunohistochemical studies of D2R, we used an affinity-purified rabbit polyclonal antiserum (Chemicon International, Temecula, CA) which is specific for D2 receptor (short form and long form) [38], [39]. For single labeling, the sections (8 sections/brain, section interval: 5) were washed three times in phosphate buffer saline (PBS, PH: 7.4) and then pre-incubated in blocking solution (3% normal goat serum and 0.3% Triton X-100 in PBS) for 1 hour. The sections were incubated with either mouse monoclonal anti-Neu-N antibody (1∶500; BD Bioscience) overnight, or rabbit polyclonal anti-D2 receptor antibody for 48 h (1∶200, Chemicon). The next day, the sections were incubated for 2 hours in biotinylated goat anti species-specific secondary antibodies (1∶200, Jackson Immunoresearch). The sections were incubated in avidin -biotin- peroxidase complex (Vectastain ABC kit, Vector Laboratories) and 3, 3′-diaminobenzidine solution (10 mg/ml, Vector Laboratories) as the chromogen.

For D2 and PV double-labeling, the sections were incubated for 48 h with rabbit anti-D2 and mouse anti-PV antibodies (1∶4000, Sigma). The next day, the sections were first incubated with biotinylated goat anti mouse secondary antibody (1∶200; Jackson Immunoresearch) for 1 hour; and after rinsing, they were further incubated in Streptavidin-Cy3 (to label PV) and Fluorescein (FITC)-conjugated goat anti-rabbit antibody (to label D2; both antibodies used at 1∶500; Jackson ImmunoResearch). The sections were finally mounted in Vectashield fluorescent mounting medium (Vectashield) and stored at 4°C.

### Quantification and cell counting

The unbiased stereology technique was used to count the number of immunopositive cells and entities in the mPFC (Stereoinvestigator, Microbrightfield). The areas of interest were defined by a contour around its perimeter at 5X objective. The actual counting of immunostained cells were done at 100X. The following analytic parameters were applied: 200×150 µm counting frame size; 20 µm optical dissector height; 1 in 5 section interval. Coefficient of error (Gundersen), m = 1 was estimated to be ∼0.03. The counting was done by a single observer blind to treatment conditions. The data obtained in each treatment group was averaged and statistically analyzed using unpaired student's *t*-test.

## Results

**Figure 1:**
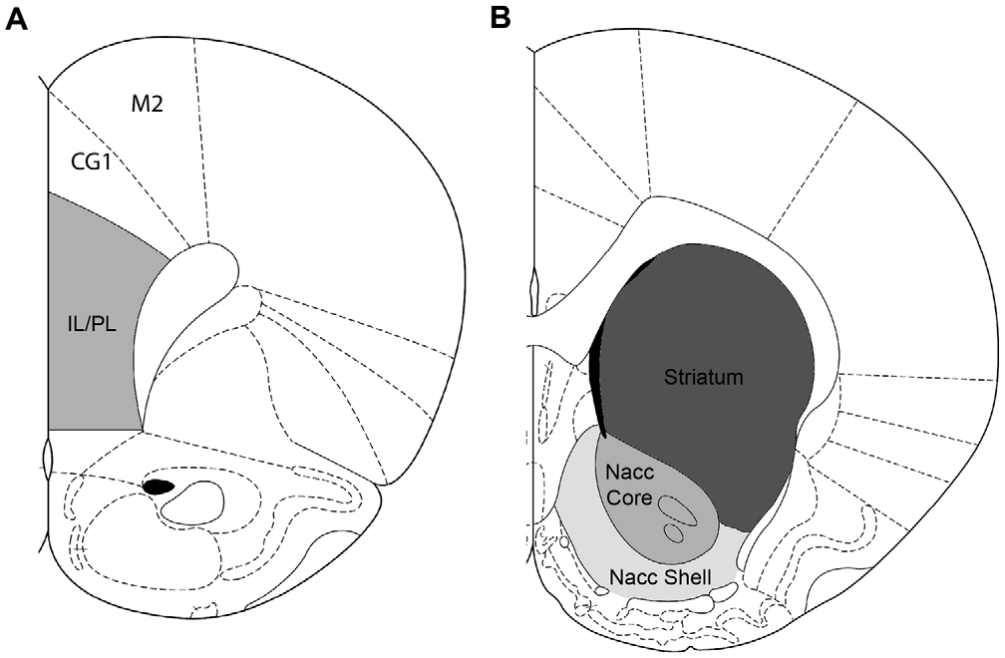
Schematic representation of the rat brain regions analyzed for the receptor expression at the levels of mPFC (A) or striatum/Nacc (B).

Schematic representation of the rat brain regions analyzed for the receptor expression at the levels of mPFC and striatum/Nacc are shown in Fig. 1A and B.

### D1 receptor binding

**Figure 2:**
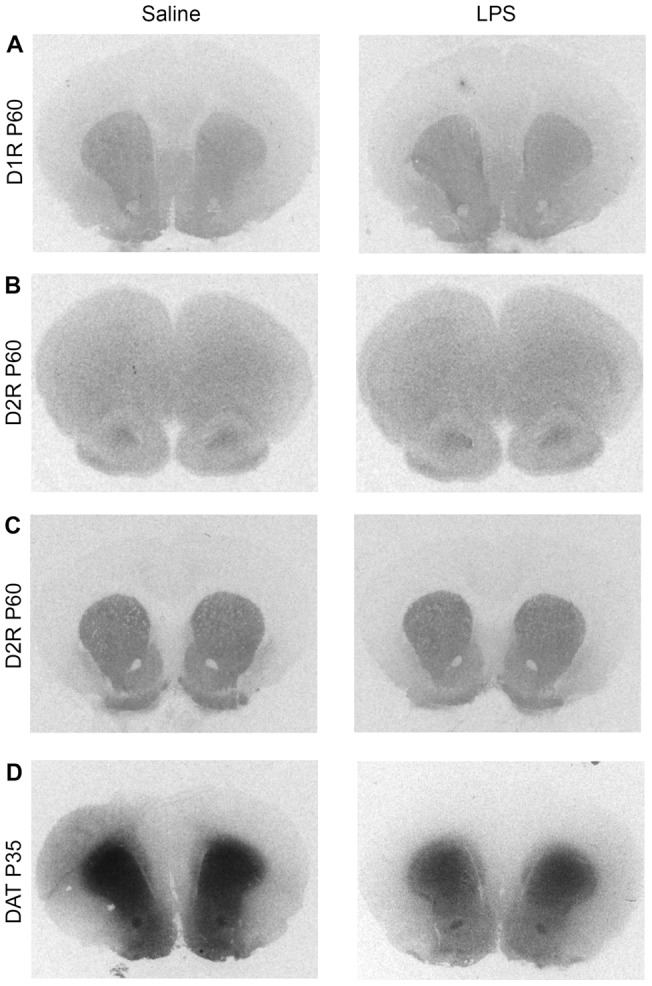
Representative autoradiograms of ligand binding to DA receptors and DA transporter (DAT) in rat brain. Left and right panels show binding in prenatal saline and LPS treatments respectively. (A), [^3^H] SCH23390 (D1-like receptor binding) in Str/NAcc; (B), [^3^H] YM-90151-2 (D2-like receptor binding) in mPFC; (C), [^3^H] YM-90151-2 in Str/Nacc; and (D), [^125^I] RTI-121 (DAT binding) in Str/Nacc. D1 and D2 autoradiograms are from animals at P60 whereas DAT binding is shown for P35 rat.

**Figure 3:**
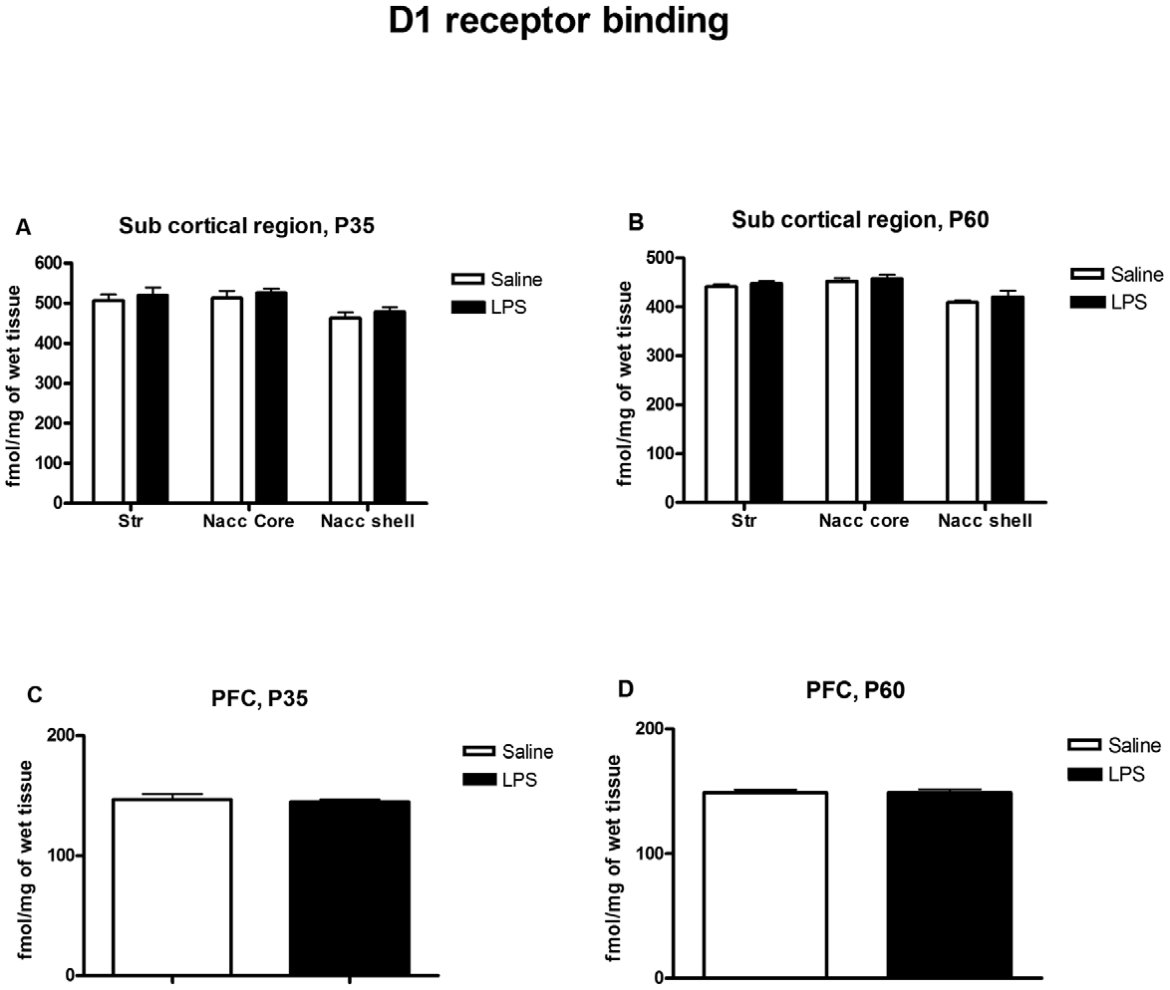
Quantitative analysis of specific binding of D1-like receptors in the prenatally saline or LPS treated offspring at P35 and P60. (A, B) are from striatal/accumbens and (C, D) are from prefrontal cortex (PFC) brain regions. No significant differences in the expression of D1-like receptors in the striatum, nucleus accumbens or prefrontal cortex were observed as a result of prenatal exposure to LPS. Values are expressed as mean±SEM. (Str; Dorsal striatum, Nacc core; Nucleus accumbens core, Nacc shell; Nucleus accumbens shell).

A representative autoradiogram of [^3^H] SCH23390 binding to D1R at striatum/accumbens levels at PD60 is shown in Fig. 2A. Comparison of the level of binding in maternal saline and LPS animals showed no significant effect of LPS treatment on D1R expression in any brain region at any time point analyzed (Fig. 3A–D). Two-way ANOVA of striatal/accumbens binding data showed a significant main effect of region at P35 and P60 (P35; *F*_(2,33)_ =  6.228, *p* = 0.0051 and P60: *F*_(2,27)_ = 17.84, *p*<0.0001) but no effect of either treatment or treatment × region interaction at any age points. Similarly, no significant difference in PFC D1R binding was found between LPS and saline groups either at P35 or P60 using student's t-test.

### D2 receptor binding

**Figure 4:**
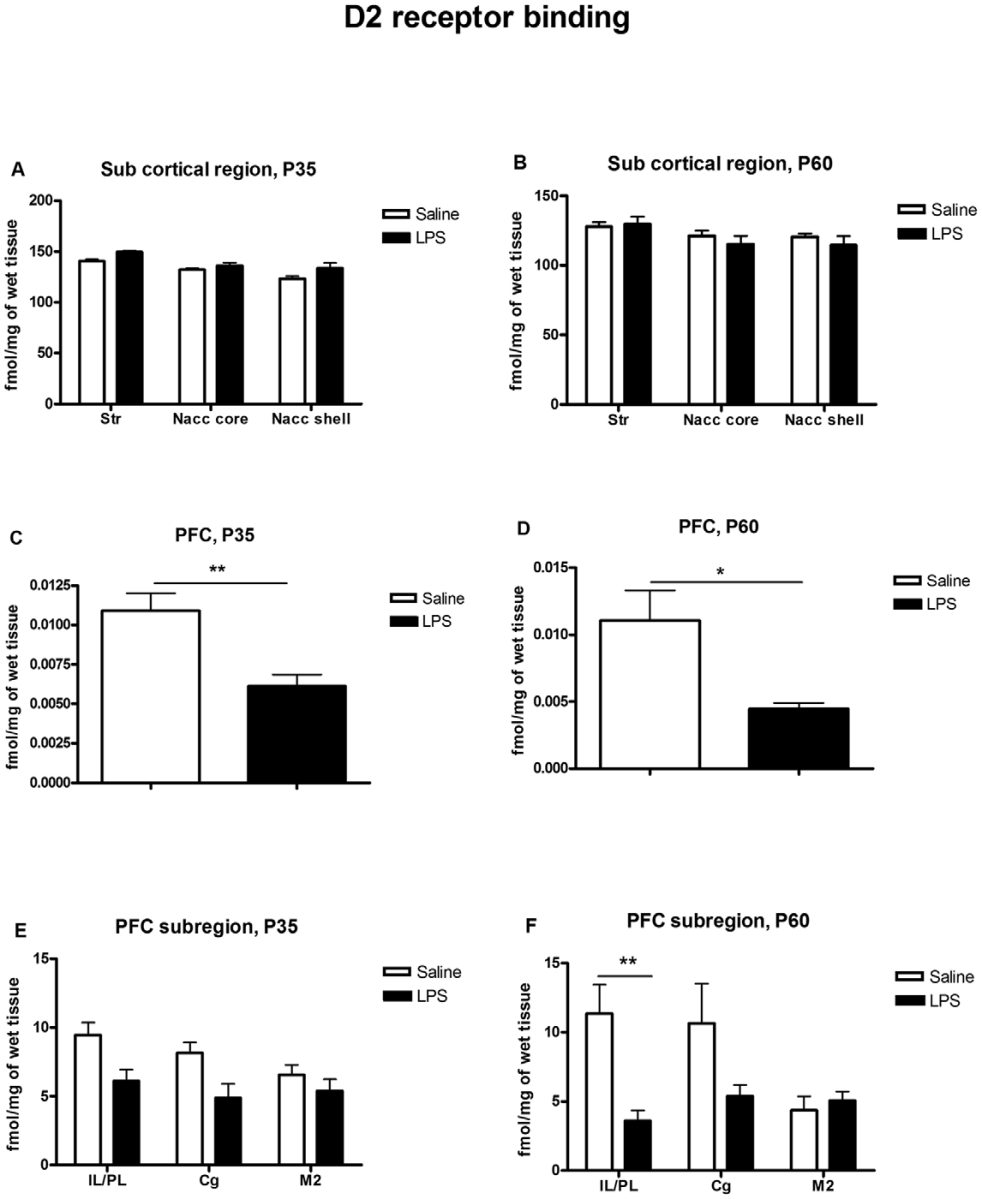
Quantitative analysis of specific binding of D2-like receptors in the prenatally saline or LPS treated offspring at P35 and P60. (A, B) are from striatal/accumbens and (C, D) are from prefrontal cortex (PFC) brain regions. Specific binding to the D2 like receptors was significantly decreased in PFC of LPS treated offspring at both P35 (**p<0.01) (C) and P60 (*p<0.05) (D). Further analysis of the data revealed that while there is no subregion-specific change in D2 binding at P35 (E), D2 like binding was significantly decreased in IL/PL subregion of mPFC at P60 (**p<0.01) which indicates age-specific changes in the expression of D2 like receptors as a result of prenatal exposure to LPS (F). Values are expressed as mean±SEM. (IL/PL; Infralimbic/prelimbic corteces, Cg; cingulate gyrus, M2; secondary motor cortex, Str; Dorsal striatum, Nacc core; Nucleus accumbens core, Nacc shell; Nucleus accumbens shell).

A representative autoradiogram of [^3^H] YM-90151-2 binding of P60 age group of animal is shown in Fig. 2 B, C. In striatum, Nacc core and shell, no significant differences in the level of D2R binding between maternal LPS and saline groups were found either at P35 or P60 (Fig. 4A and B). Analysis of D2R binding in the whole PFC by student's t-test showed a significant decrease in the level of D2R in maternal LPS-treated rats both at P35 (*p* = 0.0050) and P60 (*p* = 0.0204) (Fig. 4C, D). In order to determine whether LPS -induced changes are specific to sub- regions within the PFC, we subsequently used two-way ANOVA. At P35 we found a significant main effect of treatment (*F*_(1,15)_ = 11.18, *p* = 0.0044) but no effect of sub-region or sub-region x treatment interaction (Fig. 4E). However, P60 analysis showed a significant main effect of treatment (F_(1,24)_ = 9.850, p = 0.0045) and treatment × sub-region interaction (F_(2,24)_ = 3.686, p = 0.0402) but no significant effect of region. Post-hoc analysis revealed that the decrease in the D2R binding was limited to IL/PL regions of mPFC (** = *p*<0.01)(Fig. 4F).

### Dopamine transporter (DAT) binding

**Figure 5:**
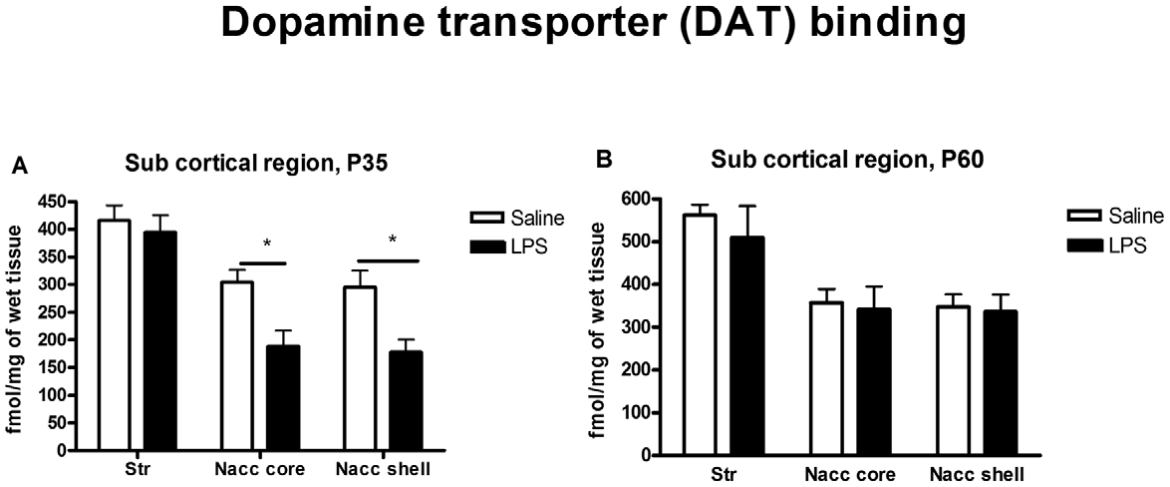
Quantitative analysis of specific binding to dopamine transporter in the prenatally saline or LPS treated offspring at P35 and P60. Due to very low level of specific [^125^I] RTI-121binding in the prefrontal cortex (PFC), quantitative analysis was not possible in this brain region. The data showed that DAT binding was significantly reduced in both nucleus accumbens core and shell at P35 (*p<0.05) in maternal LPS treated rats compared to saline treated animals (A). However we did not find any difference in the expression of DAT in the same regions at post pubertal age. All values are expressed as mean±SEM. (Str; Dorsal striatum, Nacc core; Nucleus accumbens core, Nacc shell; Nucleus accumbens shell).

[^125^I] RTI-121 binding to DAT is robust at the level of striatum/accumbens as shown in a representative autoradiogram (Fig. 2D). However, due to very low level of specific [^125^I] RTI-121binding in the PFC, we were unable to quantify DAT levels in this region. It was not surprising as in the rat PFC, very low and sparse immunoreactivity of DAT has been described previously [40]. A two-way ANOVA of P35 striatum/Nacc data yielded a significant main effect of treatment (*F*_(1,15)_ = 14.41, *p* = 0.0018), and brain region (*F*_(2,15)_ = 24.95, *p*<0.0001) but no treatment x brain region interaction Post hoc analysis showed that prenatally LPS treated rats have lower levels of DAT binding sites in Nacc, both core and shell, compared to saline control animals (*p<0.05) (Fig. 5A). At P60, we found no significant main effect of either LPS treatment or treatment x region interaction (Fig. 5B).

### Immunohistochemical analyses of D2R and PV neurons in PFC interneurons

**Figure 6:**
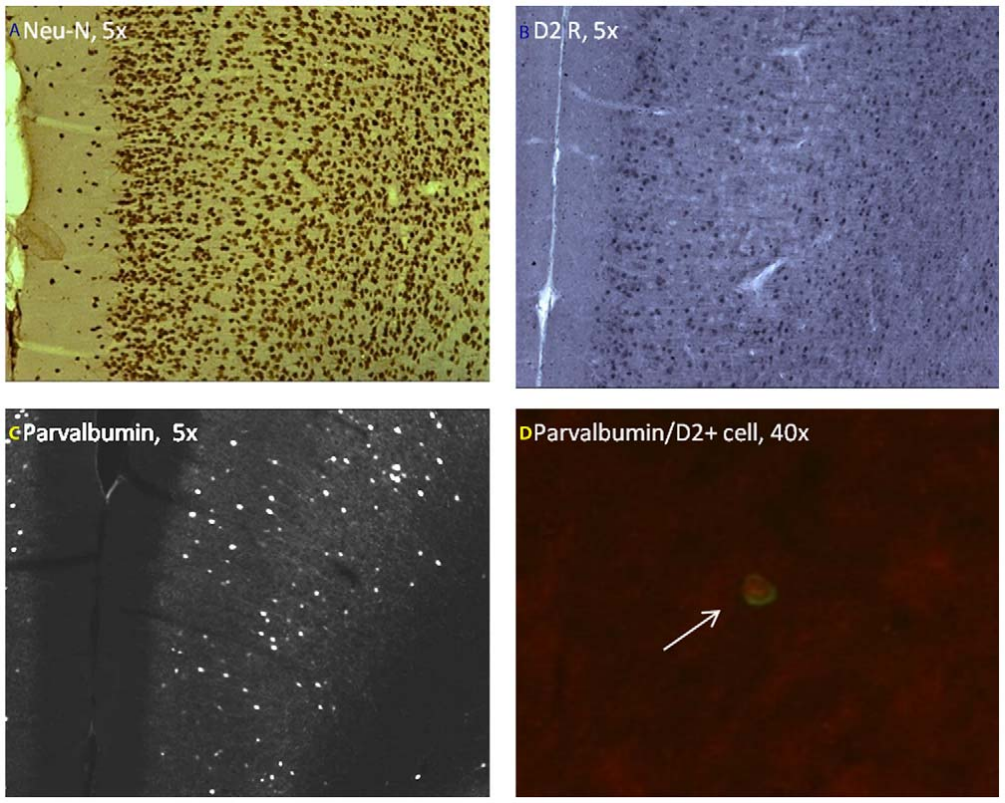
Representative immunohistochemical images of coronal brain sections of the medial prefrontal cortex (mPFC) at postpubertal age. (A) Neu-N immunolabeling (B) D2 receptor immunolabeling, (C) Parvalbumin immunolabeling and (D) Dual immunolabeling with parvalbumin (Cy3, red) and D2 receptor (FITC, green) specific antibodies.

**Figure 7:**
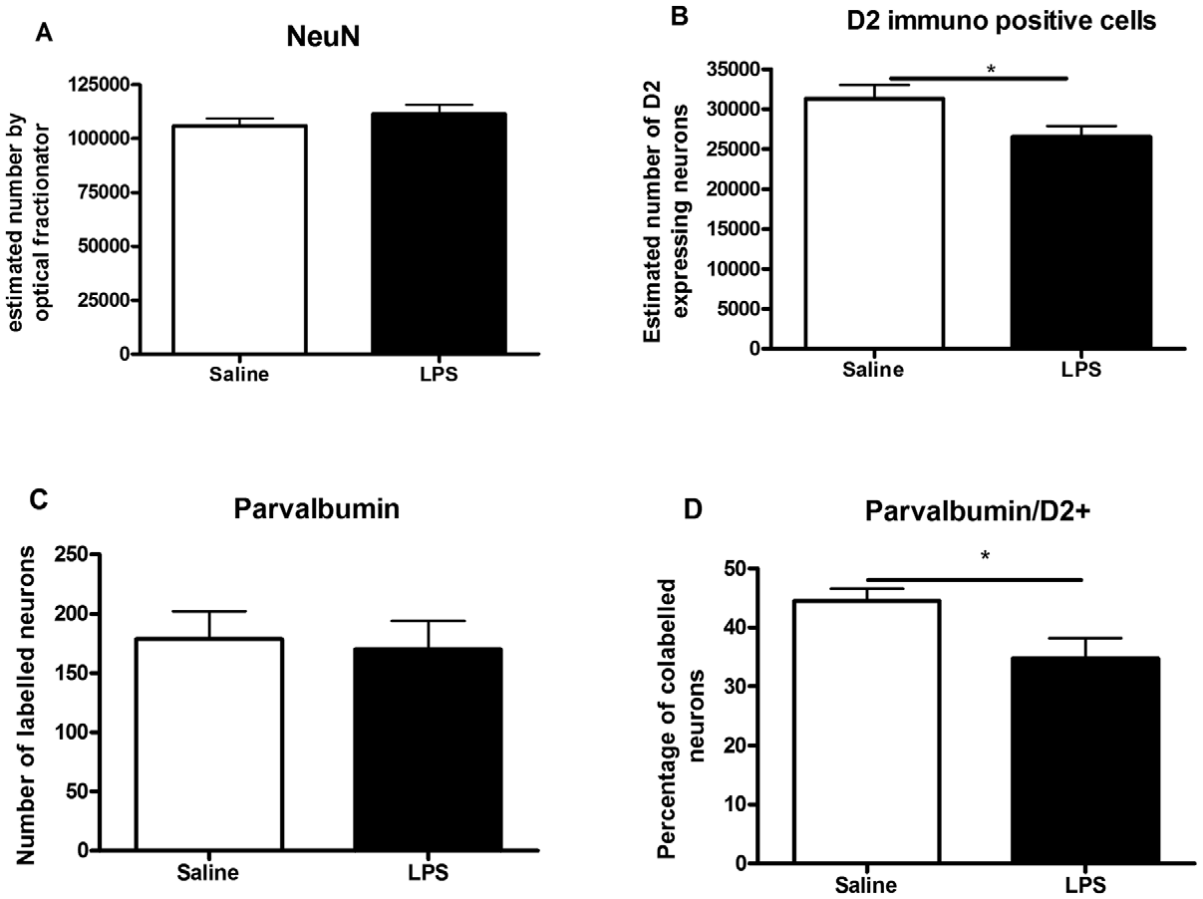
Quantitative analysis of immunolabeled cells in IL/PL subregions of medial prefrontal cortex (mPFC) at P60. (A) Total number of mature neurons (NeuN labeling) was not changed comparing maternal LPS and saline treated offspring; (B) reduction in D2-receptor immunolabeled cells in mPFC following maternal LPS challenge in comparison to saline treatment (*p<0.05); (C) number of parvalbumin positive neurons in mPFC was not changed in maternal LPS treated animals; (D) number of parvalbumin neurons which co-express D2 receptor was significantly decreased in maternal LPS animals compared to saline treated ones. (*p<0.05). All values are expressed as mean±SEM.

We first analyzed total number of mature neurons in the IL/PL PFC region of prenatal LPS and saline treated animals at P60 by Neu-N immunostaining and unbiased stereology technique (Fig. 6A). No significant difference was observed in the total number of mature neurons between prenatal saline and LPS groups (Fig. 7A). Stereological counting of D2 immunopositive cells in the IL/PL PFC at P60 showed a significant reduction in D2R expression in the LPS treated offspring compared to saline controls (*p<0.05) (Fig. 6B and 7B). This observation of decreased D2R expression is consistent with what we observed using D2 ligand autoradiography (Fig. 4F).

We next counted the total number of PV immunopositive cells, an important subpopulation of interneurons in the PFC that are implicated in schizophrenia. We found that the number of PV-immunopositive cells was comparable between prenatal saline and LPS treated animals with no significant group differences (Saline; 178.6±23.3 and LPS; 170.4±23.6) (Fig. 6C and 7C). Since D2R are also expressed in PV neurons, we counted the number of cells which were immunopositive for both PV and D2R in prenatal saline and LPS treated animals. Interestingly, here we found a significant reduction in the percentage of PV-immunopositive neurons co-expressing D2R in the IL/PL PFC of animals with prenatal LPS treatment (Saline; 44.5±2.06, LPS; 34.7±3.51,*p<0.05) (Fig. 6D and 7D).

## Discussion

The current study provides the evidence that prenatal exposure to a bacterial endotoxin (LPS) during midgestation in rats leads to significant age-dependent changes in the postnatal development of mesolimbocortical DA system in the offspring. Specifically, LPS treated offspring showed lower levels of D2 receptor in the PFC at pre and post pubertal ages (P35 and P60 respectively) as assessed by ligand autoradiography. Analysis of PFC subregions reveals that the effect is limited to the IL/PL subregions of the mPFC as the animals grow to post-pubertal age. The decreased expression of D2R was also confirmed by immunohistochemical studies. D2 immunopositive cells were found to be decreased in IL/PL mPFC at P60 while the total number of mature neurons, indicated by NeuN immunolabelling, was not altered. We also found that the number of PV-positive cells in mPFC was not altered due to prenatal LPS treatment. However, there was a significant decrease in the number of PV neurons which co-express D2R. In the subcortical regions, the only significant finding was a decrease in the level of DAT binding in the Nacc of LPS exposed animals at pre pubertal age.

Our results on mPFC D2R in GD15/16 LPS-treated rats are consistent with data reported by Meyer et al who also observed decreased D2R immunoreactivity in the mPFC of adult mice challenged prenatally with poly (I:C) at GD9 [41]. However, these authors also reported a reduction in mPFC D1R immunoreactivity in poly (I:C) treated mice. Previous findings on the expression of D1 and D2 receptors in striatal and accumbal regions in maternal poly (I:C) model appear more complex and contradictory. For example, Ozawa et al using GD12/17 poly (I:C) treatment in mice reported decreased D2 in the striatum with no changes in D1 receptors [24]. On the other hand, increased D1R in dorsal striatum and Nacc shell and increased D2R in Nacc shell has been reported in adult offspring of GD9 poly (I:C) treated mice [21]. With respect to DAT expression, our data showing decreased DAT binding in Nacc core and shell in pre-pubertal rats is consistent with those reported in poly (I:C) mice model by Vuillermot et al who also found decreased DAT immunoreactivity in P35 dorsal striatum as well as Nacc core and shell [21]. Thus, our data on maternal LPS-treated offspring suggest that while some DA-related long-term effects of maternal immune challenge may be specific to immune activating agents used, gestational time of insult or species, decreased levels of D2R in the PFC and DAT in the Nacc may be interesting common effects of maternal immune activations.

These alterations in D2 receptor and transporters could well be the substrate of DA-related behavioural changes reported in maternal infection models. For example, prenatal administration of LPS and poly (I:C) in rodents causes significant deficits in prepulse inhibition of acoustic startle and increases in amphetamine induced locomotion in the adult offspring [12], [18], [19], [23]. The significance of our D2R finding may be discussed in the context of the role of DA in mPFC-related cognitive functions such as working memory [42]–[44] which is also found to be impaired in maternal infection models [45].

To assess the cellular sites of D2R reduction in the PFC of prenatal LPS-treated animals, our focus was on PV-expressing putative interneruons. It is known that D2R is expressed in both pyramidal as well as GABAergic interneurons [46] and thus exerts direct and indirect GABA-mediated DA actions on pyramidal cells. Further, a widely-replicated postmortem finding in schizophrenia, i.e., reduction in GABA markers such as GAD67 occur in a subset of interneurons that express PV [47], [48]. In the current study, we did not find any change in the number of PV-immunopositive cells in the mPFC of LPS treated adult offspring, unlike for example in prenatal poly(I:C) model in mice where Meyer et al showed a reduction in the number of PV cells in the mPFC [13]. Our finding was a bit unexpected as decreased number of parvalbumin neurons have previously been reported in other prenatal infection models, e.g., in the hippocampus of mice offspring exposed to poly I:C at GD9 [49] as well as rat offspring exposed poly I:C at GD15 [50]. To our knowledge, our study is the first one that specifically examined total number of PV interneurons in the prefrontal cortex of rat offspring exposed to LPS during mid gestation. However, it should be pointed out that there are several key differences in the methodology and experimental design of the studies reporting changes in PV neurons and our current experiments, e.g., the timing of prenatal immune activation (GD 15/16 in our experiments vs GD 9–17), type of immune activation (LPS vs polyI:C) and animal species (rat vs mouse). In addition, our analysis of mPFC included both IL and PL subregions whereas previous studies focused on changes primarily in the IL mPFC.

Despite no significant change in the total number of PV neurons, we however, found that the percentage of the PV cells which co-express D2R was significantly decreased in the mPFC of LPS offspring. This indicates that at least a part of D2R reduction that we observed using autoradiography and immunohistochemistry occurs in the PV containing interneurons. Despite making a relatively small percentage of neuronal population in the cerebral cortex, PV containing GABAergic interneurons represent a critical regulatory element in PFC physiology and are involved in several psychiatric disorders [33], [51]. PV expressing neurons are a major class of fast spiking interneurons that play a role in PFC gamma oscillatory activity and synchronize the activity of excitatory pyramidal neurons, essential for working memory [52], [53]. D2R enhances the excitability of these fast spiking, interneurons, that mature at late adolescence [54], [55]. Interestingly, a loss of D2 modulation of fast-spiking interneurons, presumably PV-interneurons, has also been reported in the PFC of neonatal ventral hippocampus-lesioned rat model of schizophrenia [56]. Thus, our observation of a reduced D2R expression in PV cells may be another mechanism by which maternal infections affect adult PFC mediated cognitive functions.

In summary, our findings suggest that prenatal immune activation alters the development of mesolimbic and mesocortical dopaminergic system in an age specific manner. For example, decreased level of DAT expression in the nucleus accumbens is only observed at pre pubertal age in the LPS treated offspring; while at post pubertal time, we found significant decrease in D2 receptor expression in the PV interneurons of mPFC. Although extrapolation of findings from animal studies to clinical conditions must be made with caution, in the context of our data, we believe that clinical longitudinal studies on the trajectory of DA related brain changes in individuals at risk for neuropsychiatric disorders would be very informative for early detection and/or intervention purposes.
